# Effects of Four Cropping Patterns of *Lilium brownii* on Rhizosphere Microbiome Structure and Replant Disease

**DOI:** 10.3390/plants11060824

**Published:** 2022-03-20

**Authors:** Wenyue Ma, Xiaolan Liao, Chong Wang, Ya Zhang

**Affiliations:** 1 College of Plant Protection, Hunan Agricultural University, Changsha 410128, China; mawenyue2016@aliyun.com (W.M.); liaoxiaolan@hunau.edu.cn (X.L.); 2 College of Bioscience and Biotechnology, Hunan Agricultural University, Changsha 410128, China

**Keywords:** *Lilium brownii*, continuous cropping obstacle, rhizosphere soil microorganism, microbial diversity

## Abstract

Replant disease caused by continuous cropping obstacles commonly occurs in a *Lilium brownii* consecutive monoculture. To reveal the mechanisms contributing to the continuous cropping obstacles of *L. brownii*, four cropping patterns (fallow, *L. brownii*-rice rotation, newly planted *L. brownii*, and 2-year *L. brownii* consecutive monoculture) were designed, and Illumina MiSeq (16S rDNA and ITS) was utilized to detect shifts in the microbial community in the rhizosphere. Our result showed that planting of *L. brownii* significantly reduced soil pH. Consecutive monoculture of *L. brownii* can significantly decrease the diversity and abundance of soil bacteria, but markedly increase the diversity and abundance of soil fungi. Under the four planting pattern treatments, the changes in soil pH were consistent with the changes in the Shannon diversity index of soil bacterial communities, whereas we observed a negative correlation between soil pH and Shannon diversity index for fungi. The relative abundance of Lactobacillales significantly increased in soils of *L. brownii* consecutive monoculture, while Acidobacteriales, Solibacterales, and Xanthomonadales increased in soils of *L. brownii*-rice rotation and newly planted *L. brownii*. Collectively, this work aimed to elucidate the relationship between the *L. brownii* planting patterns and soil microbiome, thereby providing a theoretical basis for screening new biological agents that may contribute to resolving continuous cropping obstacles of *L. brownii.*

## 1. Introduction

Soil microorganisms are the most active and widely distributed soil organisms that participate in transforming soil nutrients, supplying nutrients for crops, and maintaining soil microecological balance and other important responses [1]. In recent years, soil microbial diversity and population structure have become popular topics in agricultural research, particularly the correlation between soil microorganisms and crops (i.e., between rhizosphere soil microorganisms and plant growth promotion, disease resistance or stress resistance); however, soil microorganisms are vulnerable to the influence of planting patterns [2,3]. China’s arable land area is small, and continuous cropping is common. However, this practice usually aggravates the occurrence of crop diseases and insect pests, affecting crop quality and leading to agricultural yield reduction in production practices [4,5].

*Lilium brownii*, a unique product of Longhui County, Hunan Province, is a perennial plant with underground bulbs. It is a kind of edible tonic and medicinal product. Bulbs are rich in a variety of proteins, carbohydrates, and saponins, with antibacterial, anti-inflammatory, sedative, anticancer, and other medicinal effects [6,7]. Longhui County is in the southwestern region of Central Hunan Province; this region has high soil fertility and a high organic content and is rich in selenium [8,9,10]. It is the origin and main producing area of *L. brownie* and thus is known as the “hometown of *L. brownie*” [11,12].

*L. brownii* is propagated asexually and cultivated perennially, and long-term continuous monoculture has resulted in severe continuous cropping obstacles (CCO) such as soil degradation, disease prevalence, yield, and quality losses. With less plantable land in major *L. brownii* producing areas, we should constantly change its planting area to other counties or provinces such as Yongzhou, Jiangxi, Yunnan, and Guizhou to meet market demand. However, differences in geographical environment and climatic conditions always affect the quality of *L. brownii* cultivation and is unfavorable for the protection of *L. brownii* in the area of origin. Therefore, a better understanding of the possible causes for the *L. brownii* CCO will help monitor and manage sustainable cultivation health in original area of lily cropping.

Crop-rotation is a useful planting management method to overcome CCO by positively impacting soil structure and plant-associated microbial communities [13]. Therefore, trying to find a complementary management practice by discussing different cropping patterns such as lily rotation with other crops is of great value to suppress *L. brownii* CCO and broaden our knowledge about *L. brownii* replant disease control. At present, there have been studies on the crop-rotation of *L. brownii*, which are related to soil-borne diseases, allelopathy of plants, and deterioration of physicochemical properties [14]. However, there are no reports on the structure and diversity of the soil microbial population. Recent reports have found that changes in bacterial diversity and keystone taxa abundances are regarded as the major factors of CCO in many plants [15]; however, the effects of the soil microbes on different lily cropping management methods and the key bacterial and fungal communities related to soil suppressiveness of lily remain unclear.

In this study, an experiment including four cropping patterns was designed, including fallow, *L. brownii*-rice rotation, newly planted *L. brownii*, two-year *L. brownii* consecutive monoculture, aiming to determine the different responses of fungal and bacterial communities in different rhizosphere soils and to explore the relationship between planting patterns and soil microbiome to provide theoretical basis to screen new biological agents that may contribute to solve CCO of *L. brownii* and guide sustainable and healthy production of *L. brownii*.

## 2. Results

### 2.1. Changes in Soil pH under Four Planting Patterns

In three different *L. brownie* growth stages, the soil pH changed differently under four cropping patterns (Table 1). The soil pH in fallow mode (RT) kept around 6.5 during the whole experimental period, while the other three *L. brownii* cultivation treatments decreased the soil pH significantly compared to RT, and the pH decreasing trend during the whole lily growth stages were in the following order: two-year consecutively monocultured plots (CC) > newly planted plots (TC) > *L. brownie*-*O. sativa*-*L. brownii* rotation plots (RC). Compared to TC and RC, the continuous cropping (CC) significantly decreased the pH, with a highest pH of 4.91 in seedling emergence period and a lowest pH of 4.33 in expanding stage. Meanwhile, crop rotation can help stability of soil pH for that the soil pH of RC (6.50) did not significantly differ from that of RT (6.69) at the seedling emergence stage, though decreased in the next two growth stages, was not as significant as TC. In addition, the variance analysis found that the cropping patterns had significant effect on soil pH (*p* < 0.01), but the growth stages affected insignificant (*p* > 0.05) (Table 2), which suggested the cropping patterns had a major impact on soil pH.

### 2.2. Overview of 16S rDNA Pyrosequencing and ITS rDNA Sequence Pyrosequencing

The microbiome structures in soils of *L. brownie* expanding stage were tested by DNA sequence pyrosequencing. A total of 74,919 effective 16S rDNA tags were generated from 12 soil samples, sample reads were mostly distributed between 350 and 399 BP (Supplementary Figure S1A; Supplementary Table S3A). Approximately 12,810, 19,815, 18,073, and 19,026 effective tags were obtained from RT, RC, TC, and CC, respectively. A total of 7745 OTUs were obtained, and 56 phyla, 178 classes, 363 orders, 564 families, and 859 genera were identified from the total sequence of 12 soil samples.

The ITS rDNA sequence pyrosequencing generated 82,154 effective tags from 12 soil samples; sample reads were mostly distributed between 200–299 bp (Supplementary Figure S1B; Supplementary Table S3B). Approximately 12,542, 16,134, 16,421, and 10,891 effective tags were obtained from RT, RC, TC, and CC, respectively. A total of 916 OTUs were obtained; 8 phyla, 31 classes, 72 orders, 135 families, and 200 genera were identified from the total sequence of 12 soil samples.

### 2.3. Bacterial and Fundal α-Diversity Analysis

The soil bacteria and fungi presented different α-diversity patterns (Table 3). For the bacteria α-diversity indices, RT showed the highest Chao1 evenness and Shannon diversity, while CC had the lowest α-diversity. RC showed a significantly (*p* < 0.05) higher α-diversity indices compared with TC and CC (Supplementary Figures S2A–S4A; Supplementary Tables S4A–S6A). The correlation between soil pH and microbial diversity (Shannon) was very high under different cropping methods (regression equation y = 1.0335x + 3.5404, correlation coefficient r = 0.9234). These results indicated that planting *L. brownie* could decrease the diversity, abundance, and quantity of bacteria in soils, and the continuous cultivation of *L. brownie* could significantly decrease soil bacterial α-diversity. Compare to newly planted plots (TC), *L. brownie*-*O. sativa-L. brownii* rotation (RC) had less impact on the soil bacterial α-diversity, the indices of which were closed to fallow mode (RT).

The α-diversity characteristics for fungal in four soil samples shared different trends with the bacteria. In terms of the fungal α-diversity indices, TC showed the lowest Chao1 evenness and Shannon diversity, while RC had the highest Chao1 index in all four samples, CC showed the highest Shannon diversity and its Chao1 value was 291.73, which was quite close to the highest Chao1 value in RC. The shannon indices of RT and RC fell in between CC and TC, in this RT was significantly higher (*p* < 0.05) than RC (Table 3; Supplementary Figures S2B–S4B; Supplementary Tables S4B–S6B). The influence of cropping patterns on the diversity, abundance, and quantity of soil fungi were more complex than that of bacteria. The continuous cropping of *L. brownii* could increase the α-diversity of soil fungi, and the remarkable higher Shannon index in CC indicated the continuous cropping may enrich fungal species in lily soils, while some of the harmful varieties may be concerned with the prevalence replant disease under this planting mode.

### 2.4. Shift in Rhizosphere Bacterial and Fungal Populations under Different Planting Patterns

**Figure 1:**
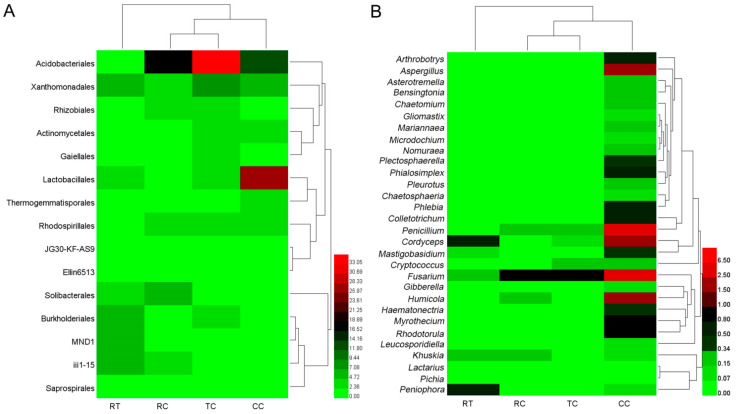
Composition of the main microorganism under four cropping patterns ((**A**): 15 bacterial orders; (**B**): 30 fungal genera).

Taxonomy analysis showed that there were 15 main soil bacterial orders in four planting soils (Figure 1A), and the relative abundance of order Acidobacteriales significantly differed among the four samples as TC > RC > CC > RT. The relative abundance of order Solibacterales was significantly higher in RC (*p* < 0.05). The relative abundance of order Lactobacillales was significantly higher in CC (*p* < 0.05).

Figure 1B showed that the relative abundance of 30 major soil fungi under the CC planting method were significantly higher (*p* < 0.05) than other treatments. The genera *Arthrobotrys, Plectosphaerella, Phialosimplex, Phlebia, Colletotrichum, Penicillium, Cordyceps, Mastigobasidium, Fusarium, Humicola, Haematonectria, Myrothecium, Rhodotorula*, and *Aspergillus* were predominant in CC, while only two genera of *Cordyceps* and *Peniophora* were predominant in RT, *Fusarium* existed in all four samples, and the abundance was as follows: CC > TC/RC > RT.

The similarity clustering of sample abundance found that RT differed from RC, TC and CC in both bacterial and fungal composition, and there were also distinct composition differences between CC and other two lily soils (TC and RC) (Figure 1). These suggested that lily planting could change microbiome structure, and the continuous lily cropping mode had a greater influence on the soil microbial communities, the changes of which may act as the major contributors for replant disease.

Table 4 showed that the abundance of *Cordyceps*, *Fusarium*, *Penicillium*, *Aspergillus*, and *Humicola* were extremely higher in CC, at 2.34%, 2.76%, 6.11%, 1.91% and 1.80%, respectively, in response to the consecutive monoculture of *L. brownii*. The differences in the abundance of the above genera were not significant in other three planting patterns, as the abundance were <1% or even zero.

**Figure 2:**
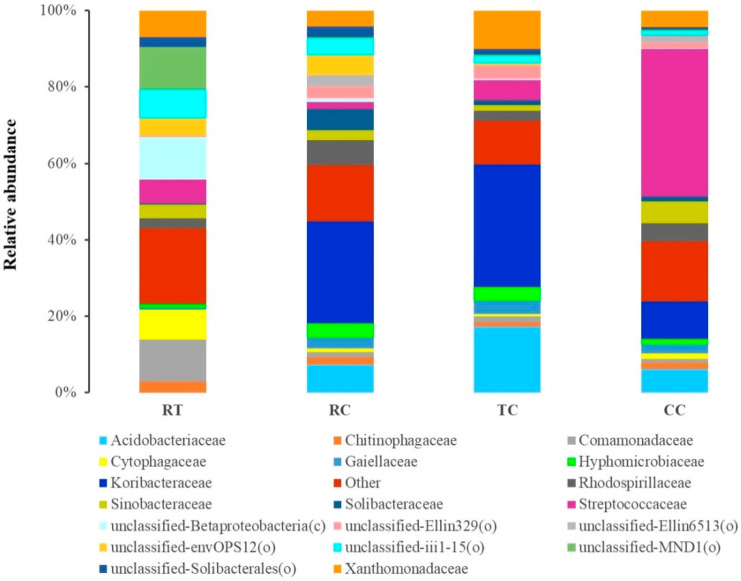
Composition of the top 20 families under four cropping patterns.

The top 20 families in four cropping soils were shown in Figure 2 (Supplemantary Table S7). There was a distinct difference in soil bacterial community between RT and *L. brownii* cultivation treatment (RC, TC and CC), which were predominated by the families Acidobacteriaceae, Koribacteraceae, Streptococcaceae, and Xanthomonadaceae. Among *L. brownii* cultivation treatment, the relative abundance of the family Streptococcaceae was significantly higher (*p* < 0.05) and that of Sinobacteraceae was significantly higher (*p* < 0.05) in unhealthy soils (i.e., CC). Compared with CC treatment, the relatively healthy soils (i.e., RC and TC) significantly increased the relative abundance of the families Acidobacteriaceae, Koribacteraceae, and Xanthomonadaceae. The paddy-upland rotation treatment RC significantly increased (*p* < 0.05) the relative abundance of the family Solibacteraceae.

## 3. Discussion

### 3.1. The Correlation between Soil pH and Rhizosphere Bacterial Diversity

Plant-associated microbiomes play important roles in promoting the plant productivity and health in natural environments [16]. Numerous studies have proven the mutual interaction between plants and their associated microorganisms [17,18,19].

This study found that consecutive monoculture *L. brownii* could decrease the relative diversities of soil bacteria, which is concordant to the results of previous studies on some other monoculture plants such as *Panax notoginseng*, *Amorphophallus konjac*, *Gossypium* spp., *Solanum melongena*, and *Lycopersicon esculentum*, the bacterial communities of rhizosphere soil under continuous cropping can destroy the original microbial population diversity and structure of rhizosphere soil [20,21,22,23,24,25]. The number of bacteria significantly decreased in the continuous cropping of *Pseudostellaria heterophylla* with the increasing of continuous cropping years [26]. Besides, we found there was a strong correlation between the changes in pH and Shannon diversity indices of bacteria communities under different cultivation treatment, which is supported by other researches that have observed that soil pH influences the relative abundance of bacteria [27]. Trivedi et al. [28] proved that soil pH and microbial diversity could impact pathogen inhibition by altering soil function. Therefore, compared with the relatively healthy soils (i.e., RT, RC, and TC), in this study, the lower pH and imbalance of microbial community structure under consecutive monoculture *L. brownii* (CC) might result in the decline in soil suppressiveness. The results also suggested that microbial diversity and the soil properties (i.e., pH) could be used as the routine detection indexes for determination of the suppressive potential of soils.

### 3.2. Changes in Microbial Composition under Four Plant Treatments

This study indicated that the planting patterns had significant influence on microbe structure and composition of the rhizosphere soil. A recent study reported that Actinobacteria was the only phylum involved in the inhibition of vascular wilt which is caused by *Fusarium* [29]. Trivedi et al. [28] then made a disease inhibition model of *F. oxysporum* and found that the abundances of Actinobacteria can be used as a major predictive marker in soil suppressiveness at the continental scale. In our study, the relative abundance of phylum Actinobacteria increased significantly (*p* < 0.05) in rhizosphere of TC than that of CC, determined that newly planted soils of *L. brownii* provided a relative strong disease suppression for *F. oxysporum* than consecutively monoculture. Many culture- and metagenomic-based studies have reported strong linkages between phylum Firmicutes and plant disease suppression [29]. It should be noted that the relative abundance of Firmicutes in our study significantly increased in soils of CC where the fungi was also abundant, implying that the taxon may be associated with early soil suppression in *L. brownii* consecutively monoculture. Members of Alphaproteobacteria and Gammaproteobacteria were also be studied in detail for their role in soil disease suppression [30,31], but Trivedi et al. [28] found that bacteria belonging to Alphaproteobacteria and Gammaproteobacteria did not act on soil suppressiveness. Similarly, at the family level, many previous studies considered an increase in the population size of Xanthomonadaceae with consecutive monoculture [32,33], whereas Campos et al. [34] claimed that Xanthomonadaceae is associated with soil suppressiveness to wheat head blight. This discrepancy might be due to different environmental conditions, host types, pathogen species, and rhizocompartments, and thus there is a need to conduct further studies on members of the phyla Alphaproteobacteria and Gammaproteobacteria and the family Xanthomonadaceae relative to *L. brownii* rhizosphere with consecutive monoculture. In contrast, the family Acidobacteriaceae has been proven to be positively correlated with soil suppression on many other crops [34], which is in line with the findings of this study, which observed an outstanding abundance of Acidobacteriaceae in RC and TC compared with CC.

### 3.3. Effects of Paddy-Upland Rotation

Crop rotation is a traditional agronomic method that has been used to regulate nutrition and reduce soilborne diseases by increasing plant diversity and reshaping the soil microbiome [35,36,37,38]. A recent study reported that under cotton-maize rotation, the diversity of the soil bacterial community increased, while the fungal community decreased [39], which is concordant to our findings on the two cropping patterns of *L. brownii*–rice rotation and consecutive monoculture *L. brownii*. Different cropping patterns significantly influences soil microbiome diversity, and the diversity of cropping plants determines the diversity of the soil microbiome community.

Moreover, it was reported that paddy-upland rotation is positively correlated with the abundance of the orders Acidobacteriales and Solibacterales [40], and some Acidobacteria subgroups were also crucial to anti-pathogens in the system [41]. In this study, the paddy-upland rotation (RC) treatment mainly increased the relative abundances of family Acidobacteriaceae, Koribacteraceae, and Solibacteraceae, which might be due to the creation of anaerobic conditions when rice is planted after *L. brownii* harvest [40,42]. Therefore, the relative lower abundance of Fusarium in RC than that in CC might be closely associated with the eminence of paddy-upland rotation in promoting bacterial diversity and modulating specific microbial composition (i.e., Acidobacteriales and Solibacterales) due to the switching between flooding and drying under anaerobic and aerobic conditions.

## 4. Materials and Methods

### 4.1. Overview of the Experimental Site

Longhui County is located at east longitude 110°38′~110°15′ and north latitude 27°10′~27°40′, which is slightly southwest of central Hunan Province in China. It has a moderate and mild climate, four seasons, and concentrated rainfall, with an annual average precipitation of 1427.5 mm. The soil type of the experimental site is red soil, the soil pH is 5.8–6.8, the texture is loose, the altitude is about 350–450 m, the annual average temperature is 16.9 °C (maximum temperature is 30 °C; minimum temperature is 8 °C), the active accumulated temperature ≥10 °C is 5312.3 °C, the frost-free period is 281 days, and the annual sunshine hours are 1539.9 h [43]. Cation exchange capacity (CEC) of the soil is 12.83 cmol/kg. Electrical conductivity (EC) of the soil is 0.45, organic matter is 22.31 mg/kg, alkaline hydrolysis nitrogen is 82.37 mg/kg, available phosphorus is 165.89 mg/kg, available potassium is 70 mg/kg.

*L. brownie* was planted in October of 2015–2018. The soil was ploughed 25-cm deep. *L. brownie* was fertilized thrice, with one base fertilizer (compound fertilizer, 750 kg/hm^2^; biological fertilizer, 3000 kg/hm^2^; alkaline fertilizer, 750 kg/hm^2^), three times top dressing. In the middle of March every year, urea (75 kg/hm^2^) was added at the seedling stage. In late May of the annual bud period, phosphate and potassium fertilizer (5 kg/mu) were added. In each year in the middle of June, at the bulbous expansion period, additional phosphate and potassium fertilizer (75 kg/hm^2^) were applied. The experimental root balls were second-generation balls of uniform size; *L. brownie* was planted in soil 7- to 10-cm deep. When planting lilies, the soil was kept moist but not waterlogged; the balls weighed approximately 100 g and were planted 15–20 cm apart. The planting density was approximately 100,000 plants/hm^2^.

### 4.2. Experimental Design

**Figure 3:**
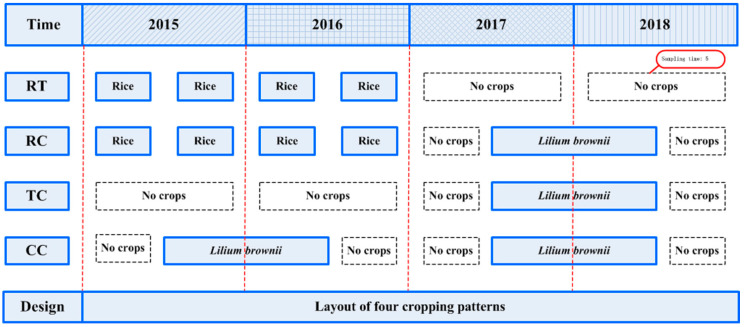
Timeframe for the field experiment and soil sampling RT: No *O. sativa* and *L. brownii* cultivation during the experimental period; RC: *L. brownie*-*O. sativa*-*L. brownii* rotation plots; TC: newly planted plots; CC: Two-year consecutively monocultured plots.

Four treatments were performed in a field previously cultivated with rice (*Oryza sativa*). Experiments included a control with no *O. sativa* and *L. brownii* cultivation during the whole experimental period (RT), *L. brownie*-*O. sativa*-*L. brownii* rotation plots (RC), newly planted plots (TC), two-year consecutively monocultured plots (CC) (Figure 3). To ensure similar environmental conditions, all four treatments had three repetitions, and the planting area of each repetition was 0.05 hm^2^. Moreover, all treatments were closely adjacent to each other and kept under the same field management during the entire experimental period.

### 4.3. Sample Preparation and Determination of Soil pH

The five-point sampling method was used for the experimental site. Seven centimeters of topsoil were removed from each point, and 200 g rhizosphere soil were collected. Five-point soil samples were mixed and quartered. One part of the soil samples was sifted through a 20-gauge mesh (aperture: 830 μm; mesh: 160) and stored at −20 °C, whereas the other part of the soil was dried naturally and sifted through a 40-gauge mesh (aperture: 380 μm; mesh: 240) at room temperature.

Ten grams of the abovementioned soil, which was dried by air and sifted through 40-gauge mesh, were added to 25 mL deionized water in a small beaker, oscillated continuously for 5 min at a rotating speed of 120 rpm, and kept for 30 min until the suspension clarified. The pH was determined by potentiometric analysis [44].

### 4.4. Extraction and Pyrosequencing of Soil Total DNA

The five-point sampling method was used to take the soil samples from RT, RC, TC and CC treatments. Seven centimeters of topsoil were removed and 50 g rhizosphere soil were collected with three repeats from each point. After decontaminization, crushing and sample mixing, 3 g soil samples were taken from each repeat and stored in the refrigerator at −80 °C for testing. Sodium dodecyl sulfate (SDS)-high salt method was used to extract and purify DNA [45]. The quality of extracted DNA was accessed by electrophoresis on 1% agarose gels, and the DNA concentrations were determined with a NanoDrop 8000 Spectrophotometer (Thermo Fisher Scientific, Waltham, MA, USA). The bacteria-specific primer 341F (5’-ACTCCTACGGGAGGCAGCA-3’)/805R (5’-GGACTACHVGGGTWTCTAAT-3’) was used to amplify the V3-V4 hypervariable region of the 16S rRNA gene [46]. The fungi-specific primer ITS1F (5’-GCTGCGTTCTTCATCGATGC-3’)/ITS2 (5’-GCTGCGTTCTTCATCGATGC-3’) was used to amplify the ITS1 region [47]. PCR reactions of the 16S rRNA gene, containing 20 μL 2 × Premix Taq (Takara Biotechnology, Dalian Co., Ltd., Dalian, China), 1 μL each primer (10 μM), and 2 μL DNA (20 ng/μL) template in a volume of 40 μL, were amplified by thermocycling: 3 min at 94 °C for initialization; 30 cycles of 20 s denaturation at 94 °C, 20 s annealing at 52 °C, and 25 s extension at 72 °C; followed by 5 min final elongation at 72 °C. PCR reactions of the ITS gene, containing 30 μL 2 × Premix Taq (Takara Biotechnology, Dalian Co., Ltd., Dalian, China), 1 μL each primer (10 μM) and 4 μL DNA (20 ng/μL) template in a volume of 45 μL, were amplified by thermocycling: 6 min at 94 °C for initialization; 30 cycles of 35 s denaturation at 94 °C, 35 s annealing at 52 °C, and 26 s extension at 72 °C, followed by 12-min final elongation at 72 °C. The Purified DNA was sent to Shanghai EOE Biotechnology Company and sequenced by the Illumina MiSeq PE300 (using paired-end reads) high-throughput method.

### 4.5. Analysis of Data

The raw reads were performed the fqtrim quality filtering, removing ambiguous, homologous, and less than 100-bp reads. Then clean data were processed on the Galaxy pipeline. USearch [48] was used to conduct chimeric sequence processing on the data after impurity removal to obtain high-quality sequences for downstream analysis. The ordered sequences were classified into operational taxonomic units (OTUs) with 97% similarity using the CD-HIT [49] classification method. The taxonomic of the 16S rRNA gene and ITS sequences representative sequences were analyzed against the Greengenes, RDP, and UNITE databases [50]. Rank-Abundance, Rarefaction, alpha diversity index with Chao1, observed species and Shannon index were calculated using the software Mothur [51]. All data were preliminarily analyzed by Excel 2010. DPS v 6.55 software was used for multiple comparisons using Duncan’s method.

## 5. Conclusions

Here, we show that continuous cropping of *L. brownii* changed the population structure and diversity of soil microorganisms. Continuous cropping of *L. brownii* may have adverse effects on the sustainable utilization of soil resources; its planting significantly reduced soil pH, and soil conditions need to be improved before lily or other crops are replanted. Moreover, the rotation of water and drought is more suitable than dry land for lily planting. Therefore, it is necessary to understand the soil microbial composition to promote the improvement of soil under continuous cropping conditions or to alleviate the obstacles of continuous cropping of *L. brownii*. Our next research will assess whether Lactobacillales and Acidobacteriales, with their significant changes in flora abundance, can be used as potential indicators for assessing soil health in continuously cropped L. brownie.

## Figures and Tables

**Table 1:** The pH values in three growth stages of *L. brownii*.

Treatment	pH		
	Seedling Emergence Period	Flourishing Period	(Bulblet) Expanding Stage
RT	6.69 ± 0.03 ^a^	6.47 ± 0.05 ^b^	6.53 ± 0.02 ^b^
RC	6.50 ± 0.16 ^b^	5.91 ± 0.05 ^c^	5.65 ± 0.11 ^d^
TC	5.25 ± 0.11 ^e^	4.65 ± 0.07 ^g^	4.61 ± 0.07 ^g^
CC	4.91 ± 0.04 ^f^	4.40 ± 0.03 ^h^	4.33 ± 0.07 ^h^

Note: Numbers with different letters in the same column are significantly different according to Duncan’s shortest significant range test (*p* < 0.05).

**Table 2:** Variance analysis of different cropping patterns (ANOVA).

Sources of Variation	Sum of Squares	Degrees of Freedom	Mean Square	F Value	*p* Value
A between factors	8.8856	3	2.9619	491.111	0.0001
B between factors	0.0055	2	0.0028	0.457	0.6386
A × B	18.4177	6	3.0696	508.978	0.0001
Error	0.1447	24	0.006		
Total variation	27.4535	35			

Note: A: Cropping pattern factor; B: Time factor.

**Table 3:** The α-diversity of rhizosphere microbial in four planting patterns.

Treatment	Bacteria		Fungi	
	Chao1	Shonnon	Chao1	Shonnon
RT	4867.00 ± 4.58 ^b^	9.95 ± 0.03 ^a^	230.50 ± 0.79 ^b^	4.53 ± 0.04 ^b^
RC	4892.00 ± 2.65 ^a^	9.90 ± 0.08 ^a^	296.47 ± 3.61 ^a^	3.83 ± 0.21 ^c^
TC	4018.33 ± 2.52 ^c^	8.53 ± 0.04 ^b^	155.90 ± 3.32 ^c^	1.96 ± 0.13 ^d^
CC	3475.00 ± 1.73 ^d^	7.65 ± 0.03 ^c^	291.73 ± 3.05 ^a^	6.02 ± 0.23 ^a^

Note: Numbers with different letters in the same column are significantly different according to Duncan’s shortest significant range test (*p* < 0.05).

**Table 4:** Population abundance of the dominant fungi genera under four cropping patterns (%).

Genera	RT	RC	TC	CC
*Arthrobotrys*	0	0.01 ± 0.002 ^e^	0	0.65 ± 0.030 ^g^
*Aspergillus*	0	0	0.01 ± 0.002 ^d^	1.91 ± 0.080 ^d^
*Colletotrichum*	0	0	0.02 ± 0.005 ^d^	0.54 ± 0.020 ^h^
*Cordyceps*	0.78 ± 0.040 ^a^	0.03 ± 0.005 ^d^	0.13 ± 0.026 ^c^	2.34 ± 0.078 ^c^
*Fusarium*	0.16 ± 0.020 ^c^	0.87 ± 0.044 ^a^	0.87 ± 0.020 ^a^	2.76 ± 0.095 ^b^
*Humicola*	0	0.22 ± 0.035 ^b^	0	1.80 ± 0.044 ^d^
*Myrothecium*	0.02 ± 0.002 ^d^	0.01 ± 0.001 ^e^	0	0.84 ± 0.046 ^f^
*Penicillium*	0	0.15 ± 0.020 ^c^	0.27 ± 0.055 ^b^	6.11 ± 0.078 ^a^
*Peniophora*	0.53 ± 0.030 ^b^	0	0	0.12 ± 0.017 ^i^
*Phialosimplex*	0	0	0	0.64 ± 0.017 ^g^
*Phlebia*	0	0	0	0.56 ± 0.017 ^h^
*Rhodotorula*	0	0.03 ± 0.001 ^d^	0	0.93 ± 0.010 ^e^

Numbers with different letters in the same column are significantly different according to Duncan’s shortest significant range test (*p* < 0.05).

## Data Availability

Not applicable.

## Supplementary Materials

The following supporting information can be downloaded at: https://www.mdpi.com/article/10.3390/plants11060824/s1, Figure S1: Histogram of the distribution of microorganism sequence lengths; Figure S2: Comparison of microorganism abundance as determined by 16S sequences and ITS sequences of different samples; Figure S3: Comparison of the number of microorganism observed species as assessed by 16S sequences and ITS sequences of different samples; Figure S4: Comparison of microbial diversity by 16S sequences and ITS sequences of different samples; Table S1: The pH values in three stages of growth of L. brownii; Table S2: Population abundance of the dominant fungi genera under four planting patterns (%); Table S3A: Histogram of the distribution of microorganism sequence lengths (bacterial); Table S3B: Histogram of the distribution of microorganism sequence lengths (fungal); Table S4A: Comparison of microorganism abundance as determined by 16S sequences of different samples (maximum value); Table S4B: Comparison of microorganism abundance as determined by ITS sequences of different samples (maximum value); Table S5A: Comparison of the number of microorganism observed species as assessed by 16S sequences of different samples (maximum value); Table S5B: Comparison of the number of microorganism observed species as assessed by ITS sequences of different samples (maximum value); Table S6A: Comparison of microbial diversity (Shannon) by 16S sequences of different samples (maximum value); Table S6B: Comparison of microbial diversity (Shannon) by ITS sequences of different samples (maximum value); Table S7: Population abundance of the dominant bacteria 20 families under four planting patterns (%).

## References

[1] Louis B.P., Maron P.A., Viaud V., Leterme P., Menasseri-Aubry S. (2016). Soil C and N models that integrate microbial diversity. Environ. Chem. Lett..

[2] Bhatia C.R. (2008). Role of microbial diversity for soil, health and plant nutrition. Molecular Mechanisms of Plant and Microbe Coexistence.

[3] Chourasiya D., Sharma M.P., Maheshwari H.S., Ramesh A., Sharma S.K., Adhya T.K. (2018). Microbial Diversity and Soil Health in Tropical Agroecosystems. Microorg. Sustain..

[4] Ryszkowski L., Szajdak L., Karg J. (1998). Effects of continuous cropping of rye on soil biota and biochemistry. Crit. Rev. Plant Sci..

[5] She S.Y., Niu J.J., Zhang C., Xiao Y.H., Chen W., Dai L.J., Liu X.D., Yin H.Q. (2017). Significant relationship between soil bacterial community structure and incidence of bacterial wilt disease under continuous cropping system. Arch. Microbiol..

[6] Hong X.X., Luo J.G., Guo C., Kong L.Y. (2012). New steroidal saponins from the bulbs of *L. brownii*. Carbohydr. Res..

[7] Ma T., Wang Z., Zhang Y.M., Luo J.G., Kong L.Y. (2017). Bioassay-Guided Isolation of Anti-Inflammatory Components from the Bulbs of L.brownii and Identifying the Underlying Mechanism through Acting on the NF-kappa B/MAPKs Pathway. Molecules.

[8] Tong Q.Z. (2009). Studies on the Germplasm Resources Evaluation and Utilization on Medical Lily in Hunan. Ph.D. Thesis.

[9] Zeng L. (2010). Current Production Situation of Longya Lilium in Shaoyang and Studies on Its In Vitro Buld Induction as well as Detoxification. Master’s Thesis.

[10] Song J.T., Lin Z.J., Zhang J.X., Meng J., Chen Z.B. (2021). Geochemical Characteristics of Selenium in Soil and Its Influencing Factors in Longhui County, Hunan Province: A Case Study of Shimen-Tantou Township. South China Geol..

[11] Li Y.F., Ming J., Liu X.Y., Wang L.G., Yuan S.X., Liu C., Wang Y., Xu L.F., Yuan Y.Y. (2013). Comparative Study on Edibility of 15 Lily Species and Varieties. Acta Hortic. Sin..

[12] Gao Z., Wang X.L., Wang B., Li R.G. (2020). Effects of fertilization on the non-virus bulb cultivation of *Lilium brownii* var. viridulum. China Cucurbits Veg..

[13] Benitez M.S., Osborne S.L., Lehman R.M. (2017). Previous crop and rotation history effects on maize seedling health and associated rhizosphere microbiome. Sci. Rep..

[14] Lu Y., Gao P., Wang Y., Li W., Cui X.W., Zhou J.M., Peng F.Y., Dai L.Y. (2021). Earthworm activity optimized the rhizosphere bacteal community structure and further alleviated the yield loss in continuous cropping lily (*Lilium lancifolium* Thunb.). Sci. Rep..

[15] Tan G., Liu Y.J., Peng S.G., Yin H.Q., Meng D.L., Tao J.M., Gu Y.B., Li J., Yang S., Xiao N.W. (2021). Soil potentials to resist continuous cropping obstacle: Three field cases. Environ. Res..

[16] Trivedi P., Leach J.E., Tringe S.G., Sa T., Singh B.K. (2020). Plant–microbiome interactions: From community assembly to plant health. Nat. Rev. Microbiol..

[17] Xue L., Ren H.D., Li S., Leng X.H., Yao H.X. (2017). Soil bacterial community structure and co-occurrence patternduring vegetation restoration in Karst Rocky desertification area. Front. Microbiol..

[18] Lundberg D.S., Teixeira P.J. (2018). Root-exuded coumarin shapes the root microbiome. Proc. Natl. Acad. Sci. USA.

[19] Acuna J.J., Jorquera M.A. (2020). Diversity, interaction, and bioprospecting of plant-associated microbiomes diversity. Diverity.

[20] Du Q., Lu D., Ma K. (2012). Effect of potato continuous cropping on soil microbial community structure and function. Ecol. Environ. Sci..

[21] Yang Q.X., Wang R.F., Xu Y.Y., Kang C.X., Miao Y., Li M.J. (2016). Dynamic change of the rhizosphere microbial community in response to growth stages of consecutively monocultured *Rehmanniae glutinosa*. Biologia.

[22] Wu J.P., Jiao Z.B., Zhou J., Guo F.L., Ding Z.L., Qiu Z.M. (2017). Analysis of bacterial communities in rhizosphere soil of continuously cropped healthy and diseased konjac. World J. Microbiol. Biotechnol..

[23] Wei Z., Yu D. (2018). Analysis of the succession of structure of the bacteria community in soil from long-term continuous cotton cropping in Xinjiang using high-throughput sequencing. Arch. Microbiol..

[24] Li H., Wang J.Q., Liu Q., Zhou Z.F., Chen F.L., Xiang D. (2019). Effects of consecutive monoculture of sweet potato on soil bacterial community as determined by pyrosequencing. J. Basic Microbiol..

[25] Zhou B.L., Xu Y., Yin Y.L., Ye X.L. (2010). Effects of different years continuous cropping and grafting on the biological activities of eggplant soil. Chin. J. Appl. Ecol..

[26] Lin M.Z., Wang H.B., Lin H.F. (2012). Effects of Pseudostellariae heterophylla continuous cropping on rhizosphere soil microorganisms. Chin. J. Appl. Ecol..

[27] Rousk J., Baath E., Brookes P.C., Lauber C.L., Lozupone C., Caporaso J.G., Knight R., Fierer N. (2010). Soil bacterial and fungal communities across a pH gradient in an arable soil. ISME J..

[28] Trivedi P., Delgado-Baquerizo M., Trivedi C., Hamonts K., Anderson I.C., Singh B.K. (2017). Keystone microbial taxa regulate the invasion of a fungal pathogen in agro-ecosystems. Soil Biol. Biochem..

[29] Cha J.Y., Han S., Hong H.J., Cho H., Kim D., Kwon Y., Kwon S.K., Crusemann M., Lee Y.B., Kim J.F. (2016). Microbial and biochemical basis of a Fusarium wilt-suppressive soil. ISME J..

[30] Weller D.M. (2007). Pseudomonas biocontrol agents of soilborne pathogen: Looking back over 30 years. Phytopathology.

[31] Pandey A., Trivedi P., Kumar B., Palni L., Man S. (2006). Characterization of a phosphate solubilizing and antagonistic strain of Pseudomonas putida (B0) isolated from a sub-alpine location in the Indian Central Himalaya. Curr. Microbiol..

[32] Jiang J.H., Song Z., Yang X.T., Mao Z.Q., Nie X.H., Guo H., Peng X.W. (2017). Microbial community analysis of apple rhizosphere around Bohai Gulf. Sci. Rep..

[33] Wu L.K., Chen J., Xiao Z.G., Zhu X.C., Wang J.Y., Wu H.M., Wu Y.H., Zhang Z.Y., Lin W.X. (2018). Barcoded Pyrosequencing Reveals a Shift in the Bacterial Community in the Rhizosphere and Rhizoplane of *Rehmannia glutinosa* under Consecutive Monoculture. Int. J. Mol. Sci..

[34] Campos S.B., Lisboa B.B., Camargo F., Bayer C., Sczyrba A., Dirksen P., Albersmeier A., Kalinowski J., Beneduzi A., Costa P.B. (2016). Soil suppressiveness and its relations with the microbial community in a Brazilian subtropical agroecosystem under different management systems. Soil Biol. Biochem..

[35] Latz E., Eisenhauer N., Rall B.C., Allan E., Roscher C., Scheu S., Jousset A. (2012). Plant diversity improves protection against soil-borne pathogens by fostering antagonistic bacterial communities. J. Ecol..

[36] Li X.G., de Boer W., Zhang Y.A., Ding C.F., Zhang T.L., Wang X.X. (2018). Suppression of soil-borne Fusariwn pathogens of peanut by intercropping with the medicinal herb *Atractylodes lancea*. Soil Biol. Biochem..

[37] Peralta A.L., Sun Y.M., McDaniel M.D., Lennon J.T. (2018). Crop rotational diversity increases disease suppressive capacity of soil microbiomes. Ecosphere.

[38] Zhou X.G., Liu J., Wu F.Z. (2017). Soil microbial communities in cucumber monoculture and rotation systems and their feedback effects on cucumber seedling growth. Plant Soil.

[39] Xi H., Zhang X.K., Qu Z. (2021). Effects of cotton–maize rotation on soil microbiome structure. Mol. Plant Pathol..

[40] Lopes A.R., Manaia C.M., Nunes O.C. (2014). Bacterial community variations in an alfalfa- rice rotation system revealed by 16S rRNA gene 454-pyrosequencing. FEMS Microbiol. Ecol..

[41] Hou P.F., Chien C.H., Chiang-Hsieh Y.F., Tseng K.C., Chow C.N., Huang H.J., Chang W.C. (2018). Paddy-upland rotation for sustainable agriculture with regards to diverse soil microbial community. Sci. Rep..

[42] Maarastawi S.A., Frindte K., Linnartz M., Knief C. (2018). Crop Rotation and Straw Application Impact Microbial Communities in Italian and Philippine Soils and the Rhizosphere of Zea mays. Front. Microbiol..

[43] Zhou R.B., He Y.S., Qu W.H., Pan Q.P., Luo Y.L. (2003). Analysis of Lily planting base in Longhui County of Hunan province. J. Hunan Univ. Chin. Med..

[44] Lu R.K. (1999). Analytical Methods of Soil Agricultural Chemistry.

[45] Zhou J., Bruns M.A., Tiedje J.M. (1996). DNA recovery from soils of diverse composition. Appl. Environ. Microbiol..

[46] Shi W., Li M., Wei G., Tian R., Li C., Wang B., Lin R., Shi C., Chi X., Zhou B. (2019). The occurrence of potato common scab correlates with the community composition and function of the geocaulosphere soil microbiome. Microbiome.

[47] Reazin C., Morris S., Smith J.E., Cowan A.D., Jumpponen A. (2016). Fires of differing intensities rapidly select distinct soil fungal communities in a Northwest US ponderosa pine forest ecosystem. For. Ecol. Manag..

[48] Edgar R.C., Haas B.J., Clemente J.C., Quince C., Knight R. (2011). UCHIME improves sensitivity and speed of chimera detection. Bioinformatics.

[49] Li W.Z., Godzik A. (2006). Cd-hit: A fast program for clustering and comparing large sets of protein or nucleotide sequences. Bioinformatics.

[50] DeSantis T.Z., Hugenholtz P., Larsen N., Rojas M., Brodie E.L., Keller K., Huber T., Dalevi D., Hu P., Andersen G.L. (2006). Greengenes, a chimera-checked 16S rRNA gene database and workbench compatible with ARB. Appl. Environ. Microbiol..

[51] Schloss P.D., Westcott S.L., Ryabin T., Hall J.R., Hartmann M., Hollister E.B., Lesniewski R.A., Oakley B.B., Parks D.H., Robinson C.J. (2009). Introducing mothur: Open-source, platform-independent, community-supported software for describing and comparing microbial communities. Appl. Environ. Microbiol..

