# Supplementary material for: Effects of Four Cropping Patterns of Lilium brownii on Rhizosphere Microbiome Structure and Replant Disease
Source: Plants (Basel). 2022 Mar 20;11(6):824. doi: 10.3390/plants11060824 (PMC8950473; doi:10.3390/plants11060824)
Supplement: Supplementary file 1 [file plants-11-00824-s001.zip › Supplementary Figure S1-S4.pdf]

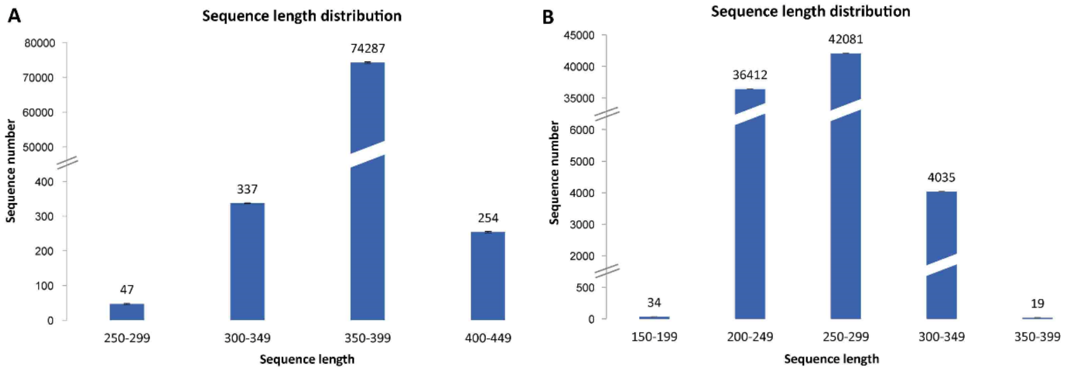


**Supplementary Figure S1.** Histogram of the distribution of microorganism sequence lengths (A: bacterial; B: fungal) Note: x-axis: bp; y-axis: band size

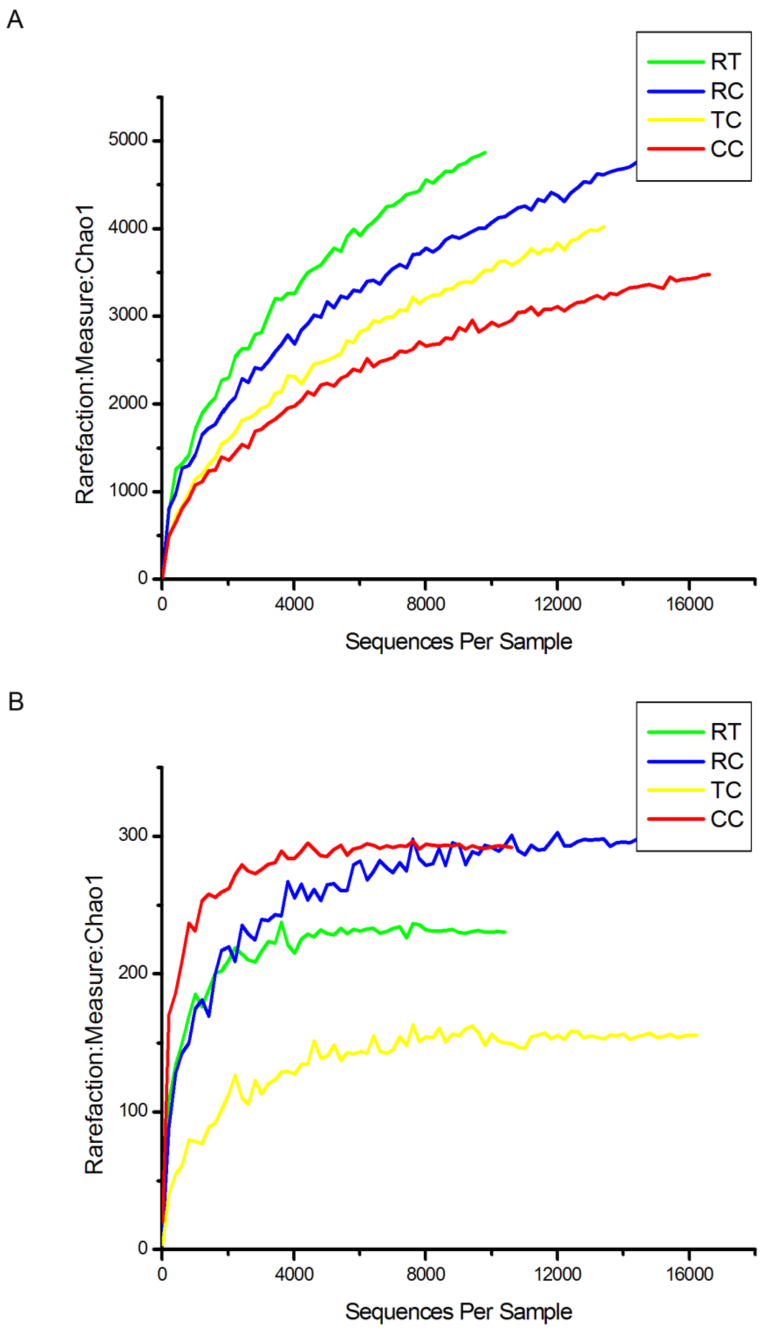


**Supplementary Figure S2.** Comparison of microorganism abundance as determined by 16S sequences and ITS sequences of different samples (A: bacterial; B: fungal) Note: x-axis: bands; y-axis: OTU

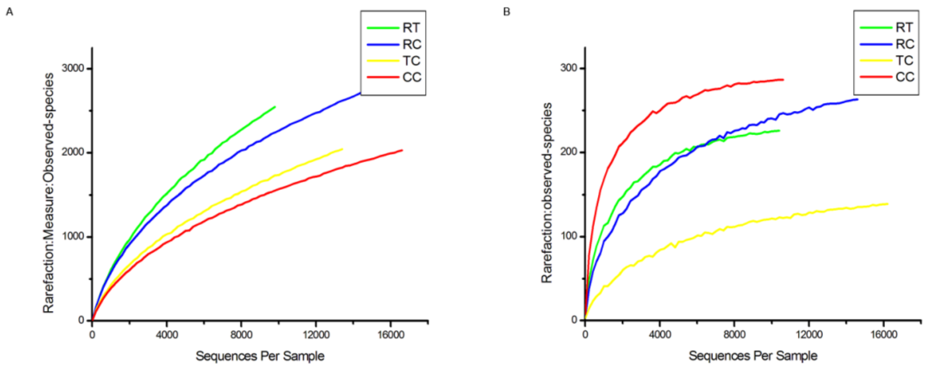


**Supplementary Figure S3.** Comparison of the number of microorganism observed species as assessed by 16S sequences and ITS sequences of different samples (A: bacterial; B: fungal) Note: x-axis: bands; y-axis: OUT

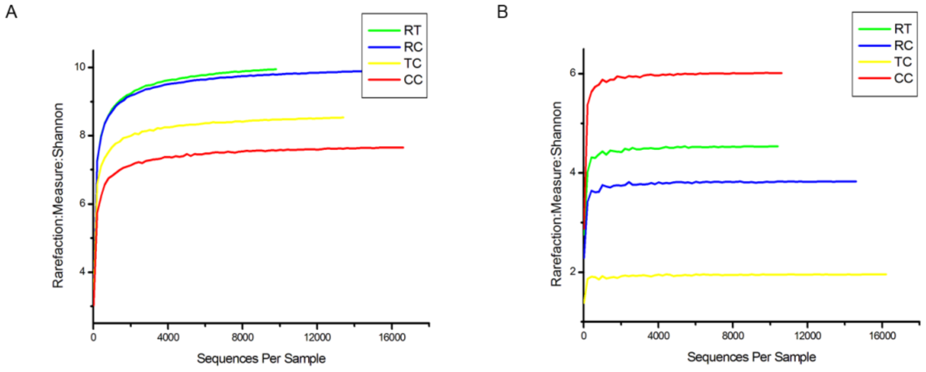


**Supplementary Figure S4.** Comparison of microbial diversity by 16S sequences and ITS sequences of different samples (A: bacterial; B: fungal) Note: x-axis: bands; y-axis: Shannon index
