# Supplementary material for: Effects of Four Cropping Patterns of Lilium brownii on Rhizosphere Microbiome Structure and Replant Disease
Source: Plants (Basel). 2022 Mar 20;11(6):824. doi: 10.3390/plants11060824 (PMC8950473; doi:10.3390/plants11060824)
Supplement: Supplementary file 1 [file plants-11-00824-s001.zip › Supplementary Table S1-S7.pdf]

Supplementary Table S1 (Anova-Data). The pH values in three stages of growth of *L. brownii*.

Treatment	pH											
	Seedling emergence period				Flourishing period				(Bulblet) Expanding stage			
	I	II	III	Average Value	I	II	III	Average Value	I	II	III	Average Value
RT	6.71	6.71	6.65	6.69 ±0.03[a]	6.49	6.51	6.41	6.47 ±0.05[b]	6.55	6.52	6.51	6.53 ±0.02[b]
RC	6.66	6.5	6.34	6.50 ±0.16[b]	5.92	5.95	5.86	5.91 ±0.05[c]	5.64	5.76	5.55	5.65 ±0.11[d]
TC	5.18	5.38	5.19	5.25 ±0.11[e]	4.66	4.71	4.58	4.65 ±0.07[g]	4.59	4.68	4.55	4.61 ±0.07[g]
CC	4.95	4.87	4.91	4.91 ±0.04[f]	4.41	4.42	4.37	4.40 ±0.03[h]	4.36	4.25	4.39	4.33 ±0.07[h]

Note: Numbers with different letters in the same column are significantly different according to Duncan's shortest significant range test ($P < 0.05$).

Table S1.1 Variance analysis of different cropping patterns (ANOVA)

Sources of variation	Sum of squares	Degrees of freedom	Mean square	F Value	p Value
A between factors	8.8856	3	2.9619	491.111	0.0001
B between factors	0.0055	2	0.0028	0.457	0.6386
AxB	18.4177	6	3.0696	508.978	0.0001
Error	0.1447	24	0.006		
Total variation	27.4535	35			

Note: A: Cropping pattern factor; B: Time factor;

Supplementary Table S2 (Anova-Data). Population abundance of the dominant fungi genera under four planting patterns (%)

Genera	RT				RC				TC				CC			
	I	II	III	Average Value	I	II	III	Average Value	I	II	III	Average Value	I	II	III	Average Value
Arthrobotrys	0	0	0	0	0.009	0.013	0.009	0.01 ±0.002^{d}				0	0.67	0.66	0.62	0.65 ±0.030^{h}
Aspergillus	0	0	0	0				0	0.008	0.011	0.011	0.01 ±0.002^{d}	1.96	1.95	1.82	1.91 ±0.080^{d}
Colletotrichum	0	0	0	0				0	0.022	0.014	0.024	0.02 ±0.005^{d}	0.52	0.54	0.56	0.54 ±0.020^{i}
Cordyceps	0.81	0.74	0.79	0.78 ±0.040^{a}	0.025	0.034	0.032	0.03 ±0.005^{d}	0.1	0.15	0.14	0.13 ±0.026^{c}	2.38	2.39	2.251	2.34 ±0.078^{c}
Fusarium	0.14	0.18	0.16	0.16 ±0.020^{c}	0.89	0.9	0.82	0.87 ±0.044^{a}	0.89	0.85	0.87	0.87 ±0.020^{a}	2.65	2.81	2.82	2.76 ±0.095^{b}
Humicola	0	0	0	0	0.26	0.2	0.2	0.22 ±0.035^{b}				0	1.77	1.85	1.78	1.80 ±0.044^{e}
Myrothecium	0.019	0.02	0.02	0.02 ±0.002^{d}	0	0.02	0.01	0.01 ±0.001^{d}				0	0.88	0.85	0.79	0.84 ±0.046^{g}
Penicillium	0	0	0	0	0.17	0.15	0.13	0.15 ±0.020^{c}	0.32	0.28	0.21	0.27 ±0.055^{b}	6.15	6.16	6.02	6.11 ±0.078^{a}
Peniophora	0.55	0.54	0.5	0.53 ±0.030^{b}				0				0	0.11	0.14	0.111	0.12 ±0.017^{j}
Phialosimplex	0	0	0	0				0				0	0.63	0.66	0.63	0.64 ±0.017^{h}
Phlebia	0	0	0	0				0				0	0.55	0.58	0.55	0.56 ±0.017^{hi}
Rhodotorula	0	0	0	0	0.04	0.03	0.02	0.03 ±0.001^{d}				0	0.92	0.94	0.93	0.93 ±0.010^{f}

Note: Numbers with different letters in the same column are significantly different according to Duncan's shortest significant range test ($P < 0.05$).

Supplementary Table S2.1 ANOVA						
Sample	Source of variation	Sum of squares	Degrees of freedom	Mean square	F Value	p Value
RT	Treatment between	2.1911	11	0.1992	995.809	0.0001
	Treatment interior	0.0048	24	0.0002		
	Total variation	2.1959	35			
RC	Treatment between	2.0534	11	0.1867	600.944	0.0001
	Treatment interior	0.0075	24	0.0003		
	Total variation	2.0609	35			
TC	Treatment between	2.1191	11	0.1926	546.383	0.0001
	Treatment interior	0.0085	24	0.0004		
	Total variation	2.1276	35			
CC	Treatment between	88.8453	11	8.0768	2901.804	0.0001
	Treatment interior	0.0668	24	0.0028		
	Total variation	88.9121	35			

Supplementary Table S3A (Anova-Data) Histogram of the distribution of microorganism sequence lengths (bacterial)

Sequence length（bp）	Sequence number			
	I	II	III	Average Value
250~299	41.00	43.00	40.00	41.33 ±1.53[d]
300~349	338.00	337.00	336.00	337.00 ±1.00[b]
350~399	74288.00	74289.00	74285.00	74287.33 ±2.08[a]
400~449	252.00	255.00	256.00	254.33 ±2.08[c]

Note: Numbers with different letters in the same column are significantly different according to Duncan's shortest significant range test ($P < 0.05$).

Supplementary Table S3.1A ANOVA					
Source of variation	Sum of squares	Degrees of freedom	Mean square	F Value	p Value
Treatment between	12346608770	3	4115536257	1371845528	0.0001
Treatment interior	24	8	3		
Total variation	12346608794	11			

Supplementary Table S3B (Anova-Data) Histogram of the distribution of microorganism sequence lengths (fungal)

Sequence length（bp）	Sequence number			
	I	II	III	Average Value
150~199	35.00	33.00	35.00	34.33 ±1.15[d]
200~249	36410.00	36416.00	36411.00	36412.33 ±3.21[b]
250~299	42081.00	42078.00	42085.00	42081.33 ±3.51[a]
300~349	4036.00	4033.00	4036.00	4035.00 ±1.73[c]
350~399	18.00	21.00	18.00	19.00 ±1.73[e]

Note: Numbers with different letters in the same column are significantly different according to Duncan's shortest significant range test ($P < 0.05$).

Supplementary Table S3.1B ANOVA					
Source of variation	Sum of squares	Degrees of freedom	Mean square	F Value	p Value
Treatment between	5247066162	4	1311766540	218627756.7	0.0001
Treatment interior	60	10	6		
Total variation	5247066222	14			

Supplementary Table S4A (Anova-Data) Comparison of microorganism abundance as determined by 16S sequences of different samples (maximum value)

Sequences Per Sample	Rarefaction Measure: chao1			
	I	II	III	Average Value
RT	4868.00	4862.00	4871.00	4867.00 ±4.58[b]
RC	4889.00	4893.00	4894.00	4892.00 ±2.65[a]
TC	4016.00	4021.00	4018.00	4018.33 ±2.52[c]
CC	3473.00	3476.00	3476.00	3475.00 ±1.73[d]

Note: Numbers with different letters in the same column are significantly different according to Duncan's shortest significant range test ($P < 0.05$).

Supplementary Table S4.1A ANOVA					
Source of variation	Sum of squares	Degrees of freedom	Mean square	F Value	p Value
Treatment between	4293688	3	1431229	153346.009	0.0001
Treatment interior	74.6667	8	9.3333		
Total variation	4293763	11			

Supplementary Table S4B (Anova-Data) Comparison of microorganism abundance as determined by ITS sequences of different samples (maximum value)

Sequences Per Sample	Rarefaction Measure: chao1			
	Ⅰ	Ⅱ	Ⅲ	Average Value
RT	229.60	230.80	231.10	230.50 ±0.79[b]
RC	299.90	292.70	296.80	296.47 ±3.61[a]
TC	153.20	159.60	154.90	155.90 ±3.32[c]
CC	288.70	294.80	291.70	291.73 ±3.05[a]

Note: Numbers with different letters in the same column are significantly different according to Duncan’s shortest significant range test ($P < 0.05$).

Supplementary Table S4.1B ANOVA					
Source of variation	Sum of squares	Degrees of freedom	Mean square	F Value	p Value
Treatment between	38923.78	3	12974.59	1527.926	0.0001
Treatment interior	67.9331	8	8.4916		
Total variation	38991.71	11			

Supplementary Table S5A (Anova-Data) Comparison of the number of microorganism observed species as assessed by 16S sequences of different samples (maximum value)

Sequences Per Sample	Rarefaction Measure: Observed-species			
	I	II	III	Average Value
RT	2548.00	2542.00	2549.00	2546.33 ±3.79[b]
RC	2797.00	2798.00	2800.00	2798.33 ±1.53[a]
TC	2042.00	2044.00	2042.00	2042.67 ±1.15[c]
CC	2028.00	2027.00	2024.00	2026.33 ±2.08[d]

Note: Numbers with different letters in the same column are significantly different according to Duncan’s shortest significant range test ($P < 0.05$).

Supplementary Table S5.1A ANOVA

Source of variation	Sum of squares	Degrees of freedom	Mean square	F Value	p Value
Treatment between	1316150	3	438716.8	78576.134	0.0001
Treatment interior	44.6667	8	5.5833		
Total variation	1316195	11			

Supplementary Table S5B (Anova-Data) Comparison of the number of microorganism observed species as assessed by ITS sequences of different samples (maximum value)

Sequences Per Sample	Rarefaction Measure: Observed-species			
	I	II	III	Average Value
RT	228.30	227.50	225.00	226.93 ±1.72[c]
RC	264.10	263.60	262.00	263.23 ±1.10[b]
TC	139.90	141.60	135.40	138.97 ±3.20[d]
CC	285.40	285.80	289.00	286.73 ±1.97[a]

Note: Numbers with different letters in the same column are significantly different according to Duncan's shortest significant range test ($P < 0.05$).

Supplementary Table S5.1B ANOVA					
Source of variation	Sum of squares	Degrees of freedom	Mean square	F Value	p Value
Treatment between	37845.98	3	12615.33	2753.928	0.0001
Treatment interior	36.6468	8	4.5808		
Total variation	37882.63	11			

Supplementary Table S6A (Anova-Data) Comparison of microbial diversity (Shannon) by 16S sequences of different samples (maximum value)

Sequences Per Sample	Rarefaction Measure: Shannon			
	I	II	III	Average Value
RT	9.98	9.96	9.92	9.95 ±0.03[a]
RC	9.93	9.81	9.97	9.90 ±0.08[a]
TC	8.55	8.49	8.56	8.53 ±0.04[b]
CC	7.68	7.62	7.65	7.65 ±0.03[c]

Note: Numbers with different letters in the same column are significantly different according to Duncan's shortest significant range test ($P < 0.05$).

Supplementary Table S6.1A ANOVA					
Source of variation	Sum of squares	Degrees of freedom	Mean square	F Value	p Value
Treatment between	11.2942	3	3.7647	1476.367	0.0001
Treatment interior	0.0204	8	0.0025		
Total variation	11.3146	11			

Supplementary Table S6B (Anova-Data) Comparison of microbial diversity (Shannon) by ITS sequences of different samples (maximum value)

Sequences Per Sample	Rarefaction Measure: Shannon			
	I	II	III	Average Value
RT	4.58	4.52	4.503	4.53 ±0.04[b]
RC	3.93	3.59	3.96	3.83 ±0.21[c]
TC	1.93	2.11	1.85	1.96 ±0.13[d]
CC	6.06	6.21	5.78	6.02 ±0.23[a]

Note: Numbers with different letters in the same column are significantly different according to Duncan's shortest significant range test ($P < 0.05$).

Supplementary Table S6.1B ANOVA

Source of variation	Sum of squares	Degrees of freedom	Mean square	F Value	p Value
Treatment between	25.5043	3	8.5014	311.305	0.0001
Treatment interior	0.2185	8	0.0273		
Total variation	25.7228	11			

Supplementary Table S7. Population abundance of the dominant bacteria 20 families under four planting patterns (%)

Taxon	RT				RC				TC				CC			
	I	II	III	Average Value	I	II	III	Average Value	I	II	III	Average Value	I	II	III	Average Value
Acidobacteriaceae	0.001	0.00001	0.001	0.00±0.00^{q}	7.43	7.27	7.41	7.37±0.09^{c}	17.29	17.26	17.26	17.27±0.02^{b}	6.18	6.26	6.17	6.20±0.05^{d}
Chitinophagaceae	2.89	2.91	2.81	2.87±0.05^{j}	1.98	1.97	1.93	1.96±0.03^{n}	1.18	1.14	1.26	1.19±0.06^{i}	1.62	1.67	1.72	1.67±0.05^{j}
Comamonadaceae	11.14	11.06	10.89	11.03±0.13^{c}	1.32	1.31	1.28	1.30±0.02^{o}	1.39	1.42	1.47	1.43±0.04^{i}	0.79	0.88	0.94	0.87±0.08^{m}
Cytophagaceae	7.89	7.83	7.92	7.88±0.05^{d}	0.86	0.92	0.93	0.90±0.04^{p}	0.66	0.57	0.63	0.62±0.05^{j}	1.38	1.42	1.51	1.44±0.07^{k}
Gaiellaceae	0.46	0.45	0.5	0.47±0.03^{n}	2.89	2.78	2.84	2.84±0.06^{l}	3.55	3.52	3.51	3.53±0.02^{f}	2.31	2.32	2.33	2.32±0.01^{h}
Hyphomicrobiaceae	0.98	0.92	0.91	0.94±0.94^{m}	3.79	3.72	3.79	3.77±0.04^{i}	3.44	3.5	3.51	3.48±0.04^{f}	1.41	1.42	1.52	1.45±0.06^{k}
Koribacteraceae	0.022	0.026	0.021	0.02±0.001^{pq}	26.69	26.72	26.66	26.69±0.03^{a}	32.09	32.14	32.17	32.13±0.04^{a}	9.92	9.98	9.81	9.90±0.09^{c}
Other	19.92	19.71	19.96	19.86±0.13^{a}	14.69	14.58	14.69	14.65±0.06^{b}	11.56	11.53	11.63	11.57±0.05^{c}	15.7	15.77	15.66	15.71±0.06^{b}
Rhodospirillaceae	2.51	2.51	2.43	2.48±2.48^{l}	6.62	6.67	6.62	6.64±0.03^{d}	2.5	2.46	2.51	2.49±0.03^{g}	4.68	4.62	4.73	4.68±0.06^{f}
Sinobacteraceae	3.62	3.64	3.62	3.63±0.01^{i}	2.64	2.65	2.57	2.62±0.04^{m}	1.48	1.52	1.51	1.50±0.02^{hi}	5.76	5.82	5.67	5.75±0.08^{e}
Solibacteraceae	0.3	0.31	0.27	0.29±0.02^{o}	5.41	5.46	5.42	5.43±0.03^{e}	1.28	1.31	1.31	1.30±0.02^{i}	1.42	1.37	1.36	1.38±0.03^{k}
Streptococcaceae	6.35	6.28	6.24	6.29±0.06^{g}	1.97	1.98	1.87	1.94±0.06^{n}	5.22	5.37	5.24	5.28±0.08^{e}	38.74	38.79	38.33	38.62±0.25^{a}
unclassified-Betaproteobacteria(c)	11.02	11.08	11.04	11.05±0.03^{bc}	0.89	0.97	0.76	0.87±0.11^{p}	0.32	0.34	0.45	0.37±0.07^{j}	0.16	0.12	0.18	0.15±0.03^{p}
unclassified-Ellin329(o)	0.38	0.32	0.33	0.34±0.03^{o}	3.16	3.18	3.11	3.15±0.04^{j}	3.33	3.31	3.42	3.35±0.06^{f}	1.32	1.45	1.41	1.39±0.07^{k}
unclassified-Ellin6513(o)	0.12	0.11	0.09	0.11±0.11^{p}	2.94	2.94	2.96	2.95±0.01^{k}	0.18	0.22	0.21	0.20±0.02^{j}	2.11	1.94	1.88	1.98±0.12^{i}
unclassified-envOPS12(o)	4.55	4.54	4.45	4.51±0.06^{h}	5.22	5.26	5.16	5.21±0.05^{f}	0.55	0.52	0.55	0.54±0.02^{j}	0.04	0.02	0.03	0.03±0.01^{p}
unclassified-iii1-15(o)	7.5	7.48	7.62	7.53±0.08^{e}	4.39	4.32	4.42	4.38±0.05^{g}	1.93	1.82	1.93	1.89±0.06^{h}	1.14	1.12	1.17	1.14±0.03^{l}
unclassified-MND1(o)	11.18	11.15	11.08	11.14±0.05^{b}	0.48	0.49	0.37	0.45±0.07^{q}	0.31	0.34	0.31	0.32±0.02^{j}	0.28	0.34	0.32	0.31±0.03^{o}
unclassified-Solibacterales(o)	2.66	2.69	2.57	2.64±0.06^{k}	2.8	2.86	2.75	2.80±0.06^{l}	1.42	1.46	1.53	1.47±0.06^{i}	0.62	0.55	0.78	0.65±0.12^{n}
Xanthomonadaceae	6.92	6.96	6.85	6.91±0.06^{f}	4.08	4.09	4.04	4.07±0.03^{h}	9.01	10.09	11.13	10.08±1.06^{d}	4.31	4.36	4.41	4.36±0.05^{g}

Note: Numbers with different letters in the same column are significantly different according to Duncan's shortest significant range test (*P < 0.05*).

Supplementary Table S7.1 ANOVA						
Sample	Source of variation	Sum of squares	Degrees of freedom	Mean square	F Value	p Value
RT	Treatment between	1573.996	19	82.8419	24446.47	0.0001
	Treatment interior	0.1355	40	0.0034		
	Total variation	1574.131	59			
RC	Treatment between	2053.702	19	108.0896	40865.64	0.0001
	Treatment interior	0.1058	40	0.0026		
	Total variation	2053.808	59			
TC	Treatment between	3450.068	19	181.5825	3124.807	0.0001
	Treatment interior	2.3244	40	0.0581		
	Total variation	3452.393	59			
CC	Treatment between	4414.155	19	232.324	33063.23	0.0001
	Treatment interior	0.2811	40	0.007		
	Total variation	4414.437	59			
